# Supplementary material for: Unraveling the crucial role of trace oxygen in organic semiconductors
Source: Nat Commun. 2024 Jan 20;15:626. doi: 10.1038/s41467-024-44897-w (PMC10799851; doi:10.1038/s41467-024-44897-w)
Supplement: Supplementary file 1 — Supplementary Information [file 41467_2024_44897_MOESM1_ESM.pdf]

Supplementary Information

Unraveling the crucial role of trace oxygen in organic semiconductors

Table of contents

Section 1. Analysis with X-ray photoelectron spectroscopy

Section 2. Temperature dependence of magnetic susceptibility

Section 3. EPR signal of purified organic semiconductors

Section 4. Theoretical calculation of oxygen doping

Section 5. Home-made *in-situ* system

Section 6. Nondestructive de-doping method

Section 7. Details of the de-doping process

Section 8. Output characteristics in the processes of de-doping

Section 9. General applicability of the de-doping and re-doping methods

Section 10. Output characteristics in the processes of re-doping

Section 11. Re-doping process under different conditions

Section 12. Proposed de-doping and re-doping routes

Section 13. Significant improvement of electron transport in *n*-type organic semiconductors

Section 14. Mobility and threshold voltage statistic for several organic semiconductors.

Section 15. Modulation of conductivity of organic semiconductor

Section 16. Interpretation of the elusive observation of subthreshold swing

Section 1. Analysis with X-ray photoelectron spectroscopy

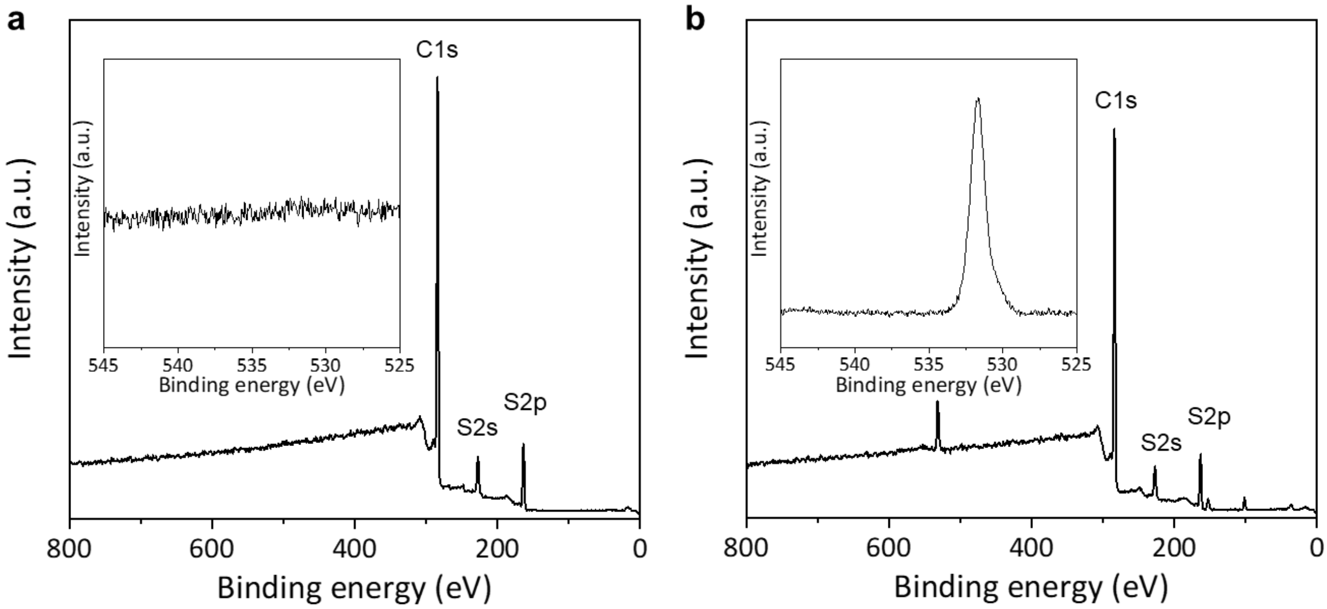

Fig. S1. X-ray photoelectron spectroscopy (XPS) of fresh deposited DNTT films. a, the fresh film. **b**, the air-exposed film. Insert is the fine spectra of oxygen (around 532 eV). The fresh DNTT film is sealed in a glass bottle in a N_2 glovebox and transferred into an X-ray photoelectron spectrometer that connects to a N_2 glovebox. XPS of the fresh DNTT film shows no oxygen-relative peak. Given the low dielectric constant of OSCs (in the range of 3 to 4), the Coulomb interactions between ionized hosts and dopants (organic radical cations and $O_2^{\bullet-}$ in this work) are on the order of several 100 meV[1], which implies that this interaction may be not broken even after sublimation (organic small molecules are generally sublimated < 400 °C). Accordingly, sublimation is not an efficient de-doping method, usually removing the physical adsorbed oxygen and resulting in chemical adsorbed trace residual.

Section 2. Temperature dependence of magnetic susceptibility

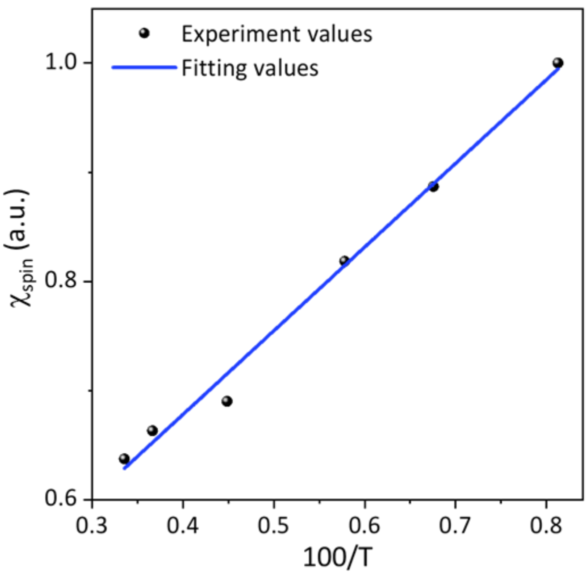

Fig. S2. Temperature dependence of magnetic susceptibility $\chi_{\mathbf{spin}}$. Susceptibility χ_{spin} is obtained by twice integrating the first-derivative of the EPR signal of DNTT. The curve of χ_{spin} versus 100/*T* shows a linear relationship, which implies that the χ_{spin} obeys the Curie law[2] $\boldsymbol{\chi}_{\mathbf{Curie}} = \left(\frac{\boldsymbol{\mu}_{\mathbf{B}}^{2}}{\mathbf{3}\boldsymbol{kT}}\right)\boldsymbol{N}_{\mathbf{spin}}\boldsymbol{g}^{\mathbf{2}}\boldsymbol{S}(\boldsymbol{S}+\mathbf{1})$, where *k*, N_{spin}, *g,* and *S* are the Boltzmann constant, total spin concentration, *g*-factor and the spin quantum number (*S* = 1/2). N_{spin} can be calculated from the Curie law by using diphenyl-1-picrylhydrazyl (DPPH) as a standard spin counting reference.

Section 3. EPR signal of purified organic semiconductors

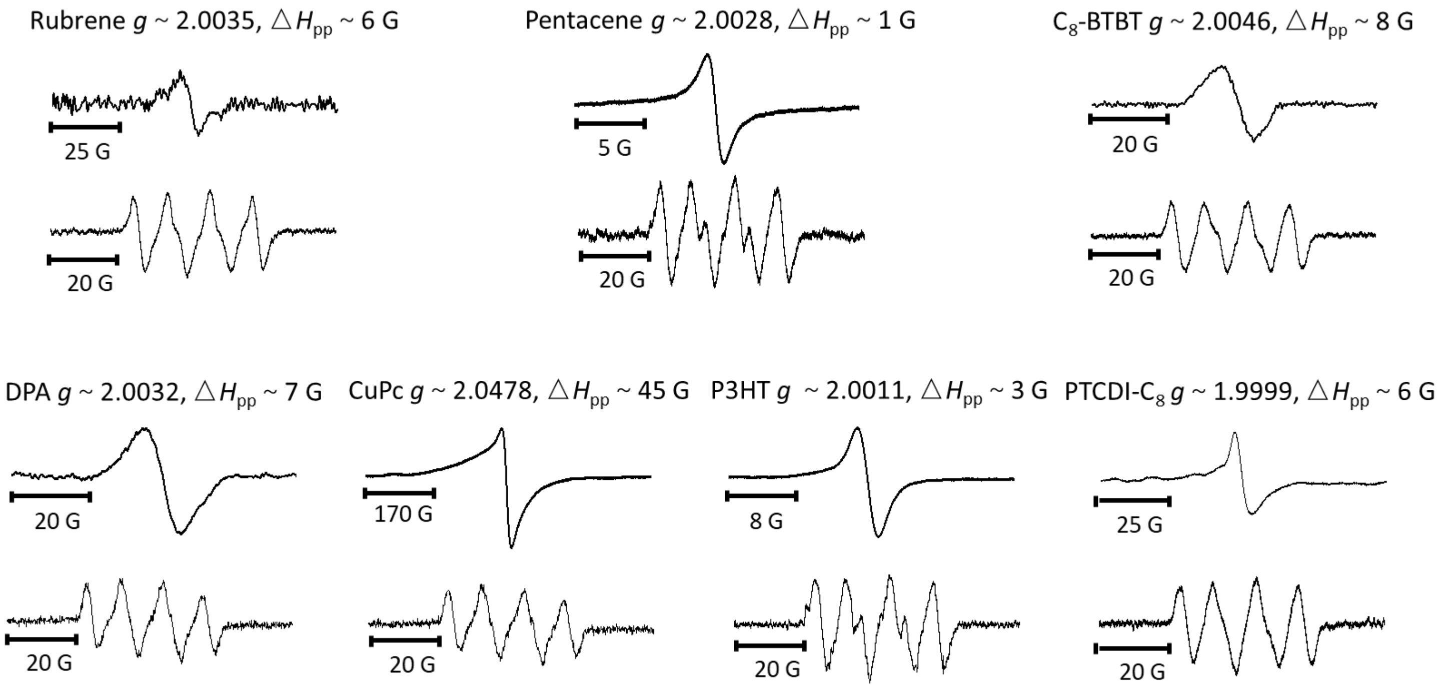

Fig. S3. EPR signal of purified OSCs. The purification of these materials (except P3HT, 99% purchased from Sigma-Aldrich) are purified by vacuum sublimation. The purified materials are loaded into glass capillaries. After sealing with silicone grease, the glass capillaries are inserted into quartz tubes for EPR testing. For the detection of O_2^-, DMPO (volume ratio of 1:100 in methanol or dimethylsulphoxide) is used as a spin trapping agent. The powders of OSCs are ultrasonically dispersed in DMPO diluent under ultrasonic dispersion for at least 30 min. The suspension liquids are sucked up with glass capillaries and sealed with silicone grease. Then, the glass capillaries are inserted into quartz EPR tubes. EPR signals of O_2^- (DMPO-OOH) are clearly observed in the suspension liquids. Some signals have only four obvious peaks, which is due to the weak intensity. The symmetrical EPR signals are observed in the powders, and show the relatively narrow ΔH_{pp} and g values close to the free electron $g_e \approx 2.0023$ (except CuPc). Considering the observation of O_2^-, these EPR signals are assigned to ORCs. The EPR signal of $CuPc^{\bullet+}$ may be obscured by this strong and overbroad signal peak of coordinated Cu^{2+} [3] and therefore not observable. These results are strong indicators of the universality of innate oxygen doping in OSCs.

Section 4. Theoretical calculation of oxygen doping

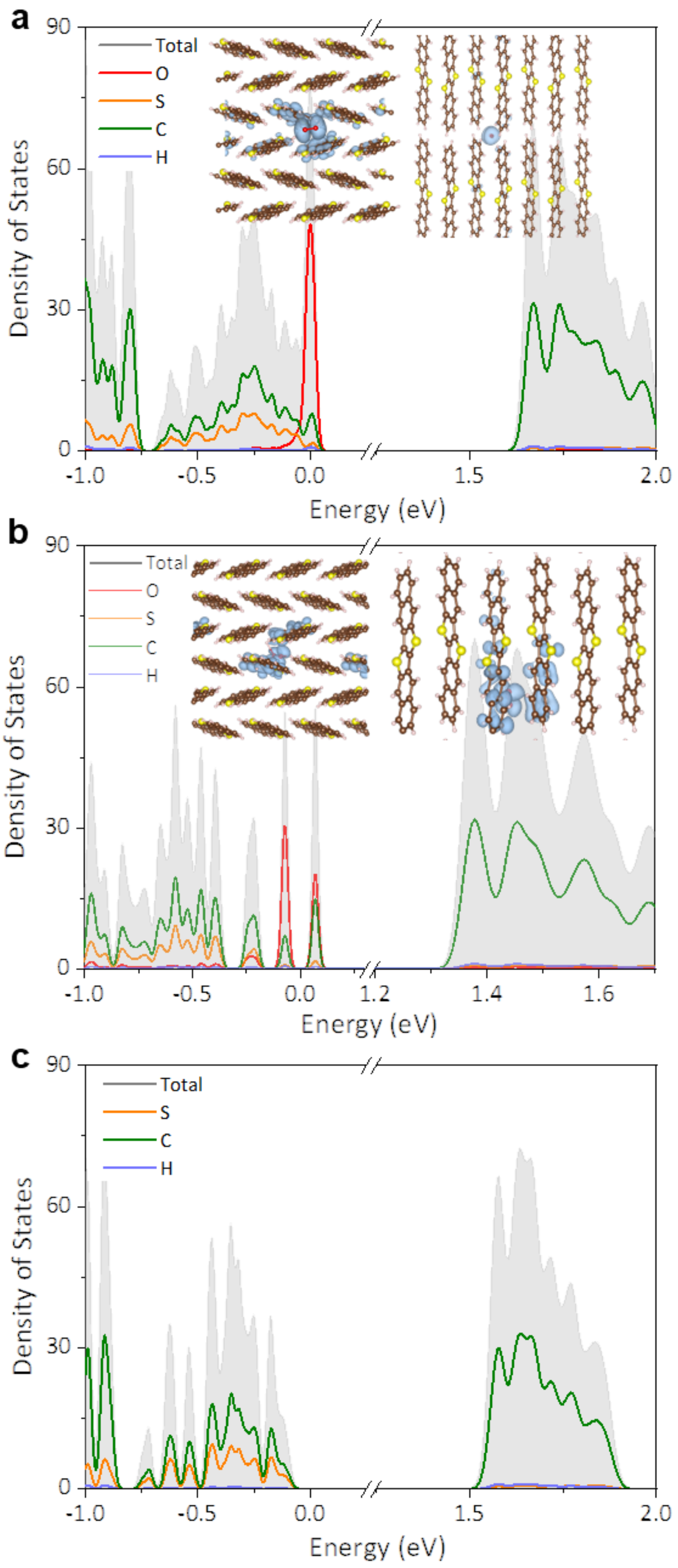

Fig. S4. Density of states (DOS) of DNTT. DNTT with oxygen incorporation into the interstitial positions of molecular interlayers (**a**) and molecular intralayers (**b**). Inserts are sketches of the occupancy sites of oxygen in the lattice of DNTT, and the yellow regions represent the spatial distribution of charge. **c**, Intrinsic DNTT. The occupancy sites of oxygen in the lattice of DNTT are investigated by first-principle molecular dynamics (FMD). According to the energy minimization

principle, oxygen prefers the interstitial positions of molecular interlayers (insert in (a)) and molecular intralayers (insert in (b)), respectively. Density functional theory (DFT) with the Perdew–Burke–Ernzerhof (PBE) exchange-correlation functional is employed to evaluate the DOS and charge spatial distribution of DNTT before and after the incorporation of oxygen. The results show that the incorporation of oxygen introduces the localized states distributed close to the edge of the HOMO and makes the extended tails shift towards higher energy levels (*i.e.*, decreasing the relative distance between E_F and the HOMO), which suggests that oxygen plays the role of acceptor doping in OSCs.

Section 5. Home-made *in-situ* system

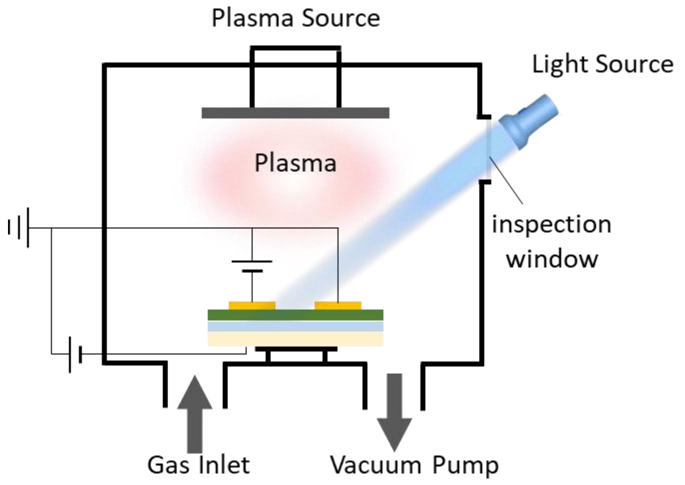

Fig. S5. Diagram of the home-made *in-situ* system. The home-made system consists of three main components: electrical measurement modules, a vacuum system (environmental control), and a plasma source. All equipment is connected to a N_2 glovebox. Two types of semiconductor device analyzers (an Agilent B1500A and a PDA FS380) are used for electrical measurement through the flange connectors. The vacuum system includes a vacuum chamber with an inspection window, pump package, and pneumatic control equipment. The plasma source is a DC glow plasma, which can be operated in a wide pressure range with an adjustable output power between 0-300 W.

Table S1. Comparation of technical parameters of several plasma source.

	DC	RF	Thermal
Plasma	Low density and energy	High density and energy	Low efficiency,
Pressure Range	10-1000 Pa	10-100 Pa	< 1 Pa
Power	0-300 W	0-100 W	0-20 W
Atmosphere	H_2, Ar, N_2 (H_2 optimum efficiency, No difference for Ar and N_2)		
Treatment Results	Ineffective (< 5 W, > 1000 Pa) **Nondestructive (< 50 W, > 50 Pa)** Destructive (> 50 W, < 50 Pa)	Destructive (all conditions)	Ineffective PS: It can be only used for ultrathin films

Firstly, we discuss the plasma source tested in this work, *i.e.*, DC plasma, RF plasma and thermal plasma (*e.g.*, we used a heated tungsten wire to ionize the thin gas in the vacuum chamber). DC source yields plasma with low density and energy, which is applicable for organic materials. RF source yields plasma with high density and energy. We had tried to use RF source at the power of 1 W to treat OFETs, but the electrical performance degraded and cannot be recovered. Thermal source is so

inefficient that it only causes a slight shift of subthreshold voltage This effect can be significant when the thickness of OSC films is very low (<5 nm).

Secondly, pressure and power both affect the plasma energy and density. At the same power, the lower pressure yields plasma with lower density but higher energy, which is prone to make a destructive effect on the OSC films. Therefore, the relative low power and high pressure are used in this work, as shown in **Table S1**. In extreme cases, the power > 200 W cause macroscopic etching within 1 min or the pressure < 10 Pa leads to irreversible performance degradation of OFETs. Regarding the processing time, it is usually less than 1min. For the case of the ultrathin films (< 5 nm), 10 s is enough. For the case of the thicker films (> 30 nm), we found that extending the processing time is a better option than increasing the power. The voltage at the anode/cathode varies with the set power and pressure. It is usually hundreds of volts with the current of tens of amperes during the plasma treatment. The plasma is not uniform in our home-built system and increases in density as the distance with electrode is closer. In this work, the samples are fixed 10 cm away from the electrode, which is the edge of the plasma glow under the power of 50 W.

Thirdly, we found that H_2 plasma is effective than Ar and N_2 under the same process parameters. We propose two possible interpretations. ① Hydrogen ion has the smaller diameter than Ar and N, which may facilitate the diffusion or penetration of the plasma. ② The reducibility of hydrogen allows it to react with oxygen to achieve a better deoxygenation effect.

Section 6. Nondestructive de-doping method

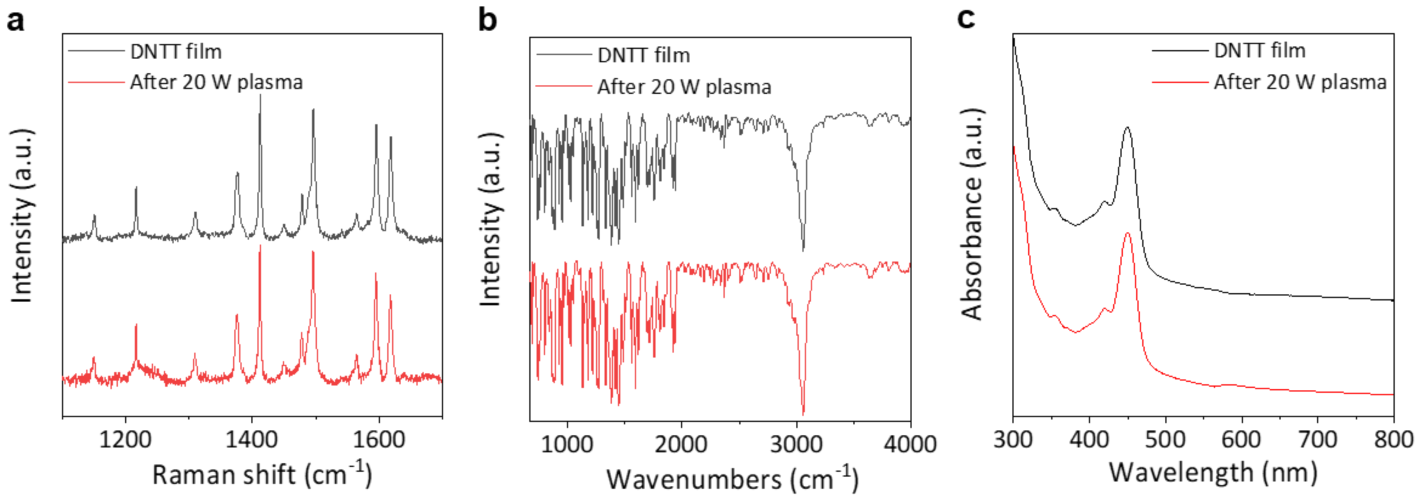

Fig. S6. Aggregate and chemical structure characterizations of the DNTT film before and after plasma treatment. a, Raman spectra. **b**, IR spectra. **c**, UV-vis absorption spectra. These results of aggregate and chemical structure characterizations show the negligible changes before and after plasma treatment, suggesting that the soft plasma treatment is nondestructive for the aggregation and molecular structures of OSCs.

Section 7. Details of the de-doping process

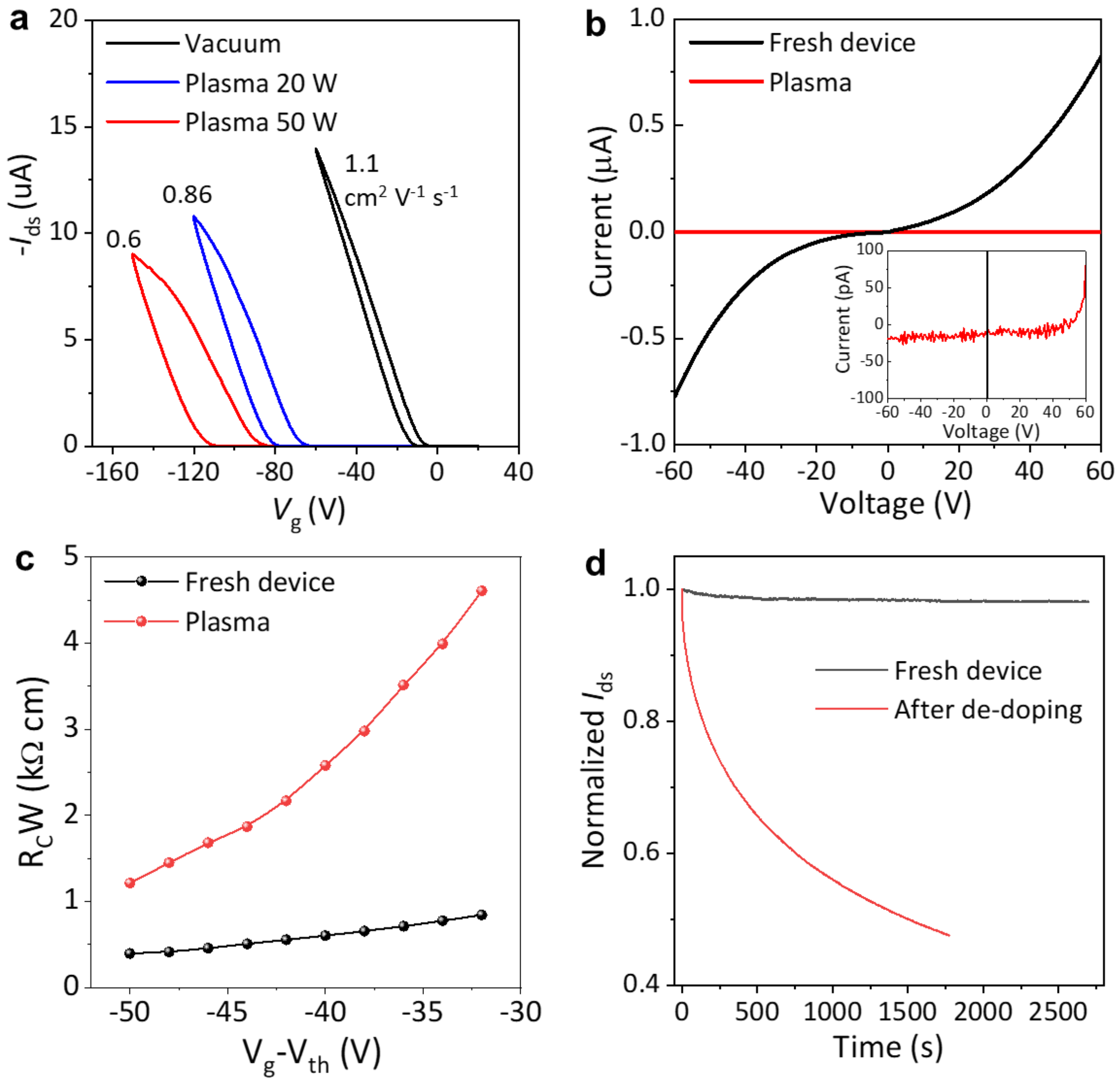

Fig. S7. a, Transfer curves in linear region of the DNTT OFET during de-doping process. V_{ds} = -5 V **b**, Conductivity of the OFET by two-terminal measurement before and after de-doping treatment. Insert shows the partially enlarged details. **c**, Width-normalized contact resistance (RW) plotted as a function of the gate-overdrive voltage (V_g-V_{th}) extracted by the transmission line method. **d**, Bias stress test of the DNTT OFET before and after de-doping treatment (plasma 10 W, 1 min under H_2 200 Pa).

Mobility (μ) in the linear region is extracted by field-effect method:

$$I_{ds} = \frac{W}{L}\mu C_i(V_g - V_{th})\, V_{ds} \qquad (1)$$

where W and L are width and length of the conducting channel, respectively, and C_i is the unit-area capacitance of the dielectric layer. After plasma treatment, the hysteresis of curves obviously increases and non-linear occurs under high overdrive voltage, which indicates the increase of density of trap states. It is because the pre-emptying effect of oxygen doping is removed by plasma treatment. Although the threshold voltage shifts significantly, no order of magnitude drop is in on-current

observed under the similar overdrive voltage, suggesting no obvious change in mobility. In fact, the extracted mobility does show only a 50% decrease. The slight decrease can be explained by the increase of trap density according to MTR model[4]. This result indicates that mobility is a nature of materials and not significantly influenced by doping even considerable trap states inherent in OSCs.

The gate voltage dependent contact resistances of the OFET before and after plasma treatment are extracted by the transmission length method (TLM). According to the experimental data, the contact resistance increases by ~2 times after de-doping at V_g - V_{th} = 50 V, and the total resistance increases by ~4 times under the same overdrive voltage. It suggests that the contribution of doping on channel and contact resistance is roughly the same under the high overdrive voltage. However, the doping contribution on channel will be much greater than that on contact resistance under overdrive voltage. For example, the contact resistance increases by ~4.75 times after de-doping at V_g - V_{th} = 32 V, but the total resistance increases by ~ 280 times. It indicates that trace oxygen doping is more prominent under low density of gate-induced carriers.

The stability of the OFETs before and after de-doping is examined. The bias test is the most representative method to assess the stability of devices. In this test, V_{ds} and V_{gs} are set to – 60 V. As shown in Fig. S7d, the fresh DNTT OFET show negligible decrease in current within 3000 s whereas the current rapidly decrease within 2000 s after de-doping treatment (plasma 10 W, 1 min under H_2 200 Pa). The strong bias stress indicates the significant increase in trap density, which is attributed to trace oxygen de-doping resulting in the disappear of trap pre-empty effect. This result demonstrate that trace doping is important for device stability.

To explore details on the energy distribution of the trapping states within the bad gap, *i.e.*, the trap DOS function, Kalb's method[5] is used to quantitatively determine the trap DOS. This method assumes that the semiconductor layer is homogeneous and accounts for the initial band bending by calculating the gate–source voltage above the flat band voltage, *i.e.*, $U_g = |V_g - V_{FB}|$. V_{FB} is assumed to be the turn-on voltage estimated from the transfer curve. The function V_0 (U_g) is then obtained by numerically solving the following equation:

$$\exp\left(\frac{eV_0}{kT}\right) - \frac{eV_0}{kT} - 1 = \frac{e}{kT}\frac{\varepsilon_i d}{\varepsilon_s l \sigma_0}\left[U_g\sigma(U_g) - \int_0^{U_g}\sigma(\widetilde{U_g})d\widetilde{U_g}\right] \quad (2)$$

where ε_i and ε_s are the relative permittivity of the dielectric and semiconductor layers, and l and d the thickness of the dielectric and semiconductor layers, respectively, $\sigma(U_g)$ is the field-effect conductivity evaluated from the linear regime transfer characteristics (ID vs. VGS curve) using equation $\sigma(U_g) = LI_D/WV_D$ and σ_0 is the conductivity at flat band. Then, the total carrier density can be calculated by V_0 (U_g),

$$p(V_0) = \frac{\varepsilon_0 {\varepsilon_i}^2 d}{\varepsilon_s l^2 e} U_g \left(\frac{dV_0}{dU_g}\right)^{-1} \qquad (3)$$

Finally, the trap DOS was found to be

$$N(E) \approx \frac{1}{e}\frac{dp(V_0)}{dV_0} \qquad (4)$$

in which $E = eV_0$ is the energy relative to the Fermi energy E_F [6].

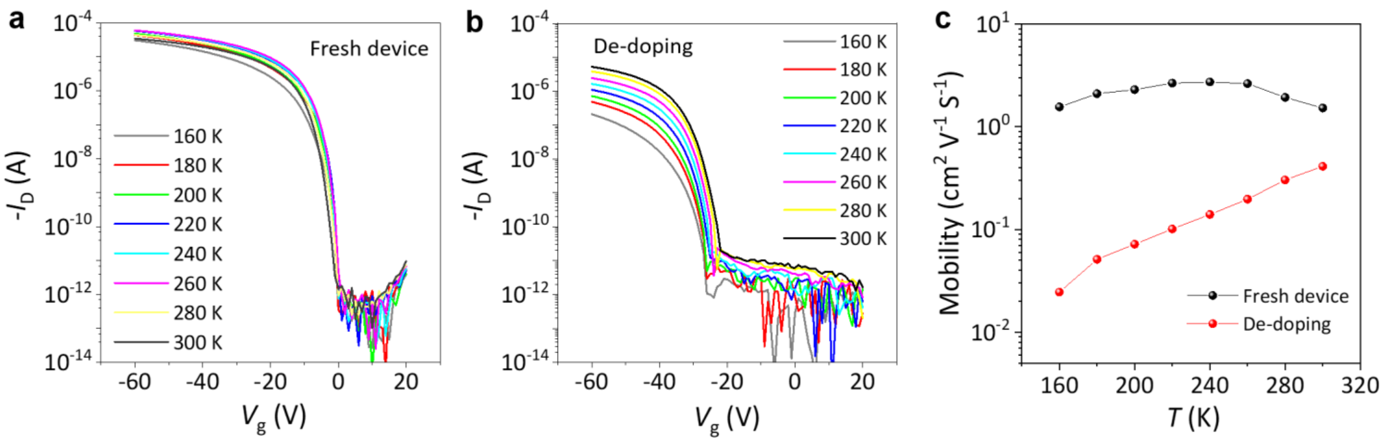

Fig. S8. Temperature-dependent measurements of the OFET before and after de-doping. Transfer curves of the fresh device (**a**) and de-doped device (**b**). **c**, Temperature-dependent mobilities of the fresh and de-doped devices.

To further understand the role of oxygen doping in charge transport, the low-temperature measurements for the DNTT OFET before and after de-doping are performed (Fig. S8). As the temperature decreases, the on-state current of the fresh device increases at first and then decreases with negligible threshold voltage shift while the de-doped DNTT OFET shows obvious degradation in both on-state current and threshold voltage. The mobilities of the fresh and de-doped OFETs suggest different temperature dependence; the former increases at first and then decreases with the decrease of temperature, according with the feature of MTR model[4], and the latter monotonically decreases, which is a typical hopping behavior. These results match exactly with our insight of oxygen pre-empty effect. Mobility is governed by the time that a carrier transports within extended band (τ) and spends within a trap (τ_t), *i.e.*, $\mu_{eff} = \mu_0[\tau/(\tau+\tau_t)]$. For the case of doped state, oxygen pre-empties the trap states. The OFET shows a band-like mobility due to $\tau >> \tau_t$ in the relative high temperature range, but a thermal activated mobility because τ_t increases in the relative low temperature range. For the case of de-doped state, oxygen pre-emptying effect is removed. A lot of trap states make $\tau_t >> \tau$ in whole temperature range, resulting in a typical hopping mobility.

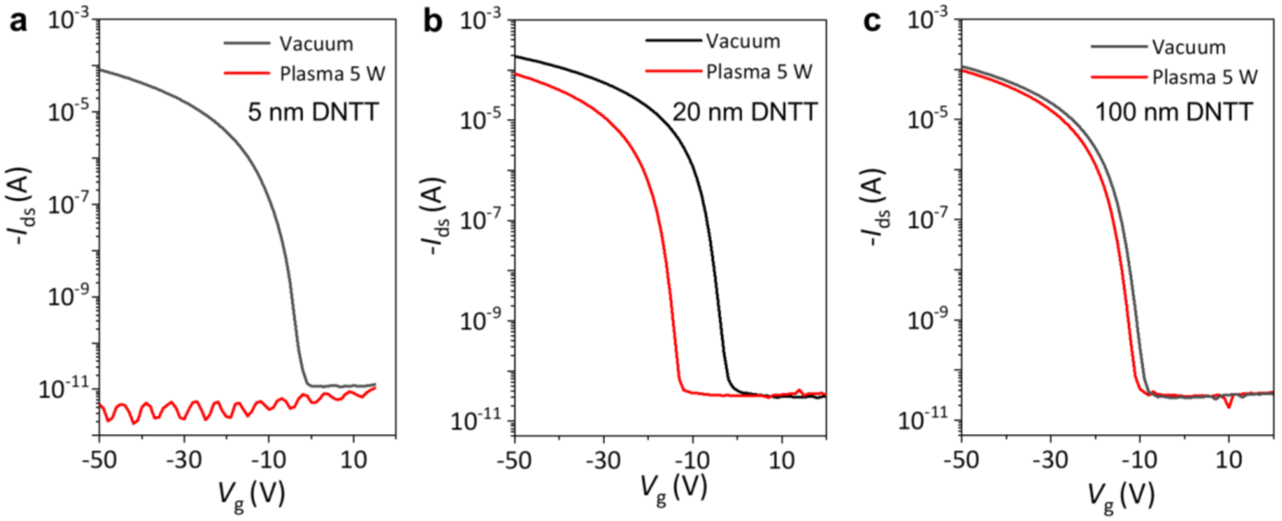

Fig. S9. De-doping process of varying thickness of DNTT OFETs. The films with thickness of 5 nm (**a**), 20 nm (**b**), and 100 nm(**c**) are used in this test.

The effect of varying thickness of DNTT OFETs on the de-doping process is investigated. The 5 nm, 20 nm, and 100 nm DNTT films were used, and the de-doping conditions are set as 5 W, 1 min. As shown in **Fig. R12**, the transfer characteristics of 5 nm film completely disappeared, while 20 nm and 100 nm films exhibited the shift of threshold voltage and the shift decreased with the increase of thickness. This result suggests that the thickness of organic films significantly hinders the de-doping effect of plasma, which could be due to the penetration limitation of plasma in crystalline film.

Donor-like traps. The static/dynamic disorder in OSC devices causes trap states distributed in the forbidden band[7-9]. Some trap states distributed below the Fermi level (E_F) are generally occupied by electrons and show electrical neutrality. When E_F moves down across these trap states, they will donate electrons and thus are positively charged (*i.e.*, trap states are emptied); a process that looks just like capturing hole carriers. These states are called as donor-like traps[10,11]. Donor-like traps can pin E_F. E_F cannot be moved to the HOMO until these traps are "filled". Mobility is governed by trap states as expressed by the multiple trapping and release (MTR) model[4],

$$\mu_{eff} = \mu_0(\tau/(\tau + \tau_{tr})) \quad (5)$$

where μ_{eff} and μ_0 are the effective and intrinsic mobility, respectively, and τ and τ_{tr} are the time that a hole carrier propagates between the traps, and spends within a trap, respectively.

Metal/semiconductor interface. The charge transport is inevitably affected by charge injection at the metal/semiconductor interfaces. A Schottky junction generally forms at metal/semiconductor interfaces in organic photoelectric devices because of mismatched energy levels[12,13], interface dipoles [14,15], and surface states[16], *etc*.. Carriers are injected into OSCs across junctions mainly by thermionic emission (TE) and thermionic field emission (TFE) at room temperatur[17,18]. The former is governed by the height of the Schottky barrier (E_B), which is modulated *via* the lowering induced by image force,

$$\Delta E_{\mathrm{B}} = \left[\frac{q^3 N_A |\varphi_s|}{8\pi^2 \varepsilon_s^3}\right]^{1/4} \quad (6)$$

where ΔE_{B} is the E_{B} lowering, ε_{s} and N_{A} are the relative dielectric constant and acceptor density (*i.e.*, hole carrier density) of OSCs, respectively, φ_{s} is the surface potential. And the latter is highly dependent on the width of the depletion layer (W_{D}). The width of the depletion layer (W_{D}) can be described as[10]:

$$W_{\mathrm{D}} = \sqrt{\frac{2\varepsilon_{\mathrm{s}}}{qN_{\mathrm{A}}}\left(\varphi_{\mathrm{bi}} - V - \frac{kT}{q}\right)} \quad (7)$$

where φ_{bi} is the built-in potential.

Oxygen doping. Oxygen acts as an acceptor in OSCs and its states distribute along the edge of the HOMO. Oxygen doping pre-empties the donor-like traps and thus eliminates the E_{F} pinning, which renders the E_{F} close to the edge of the HOMO, leading to mobile hole carriers in the HOMO. According to MTR model, mobility should increase, benefiting from the pre-emptying of traps. The effect of oxygen doping on charge injection is reflected in W_{D}. In terms of equation (7), the increased carrier density by oxygen doping reduces W_{D}, which facilitates carrier injection through both TE and TFE. Accordingly, the conductivity of the OSC is improved.

De-doping. After plasma treatment, oxygen doping is eliminated, shifting E_{F} away from the HOMO and broadening the depletion layer. As a result, the hole carriers decrease sharply and TFE is suppressed, which causes a decrease in conductivity and off-state current. Furthermore, the de-doping makes the pre-emptied donor-like trap states occupied, and the carrier transport is strongly impeded by the trapping effect. Subsequently, the subthreshold swing deteriorates and mobility decreases. At this point, even if a large gate voltage is applied, E_{F} is not close enough to the HOMO due to the larger energy difference and the E_{F} pinning effect of the traps, and hence the threshold voltage dramatically increases. The above analyses qualitatively explain the decline and the ultimate disappearance of *p*-type characteristics.

Section 8. Output characteristics in the processes of de-doping

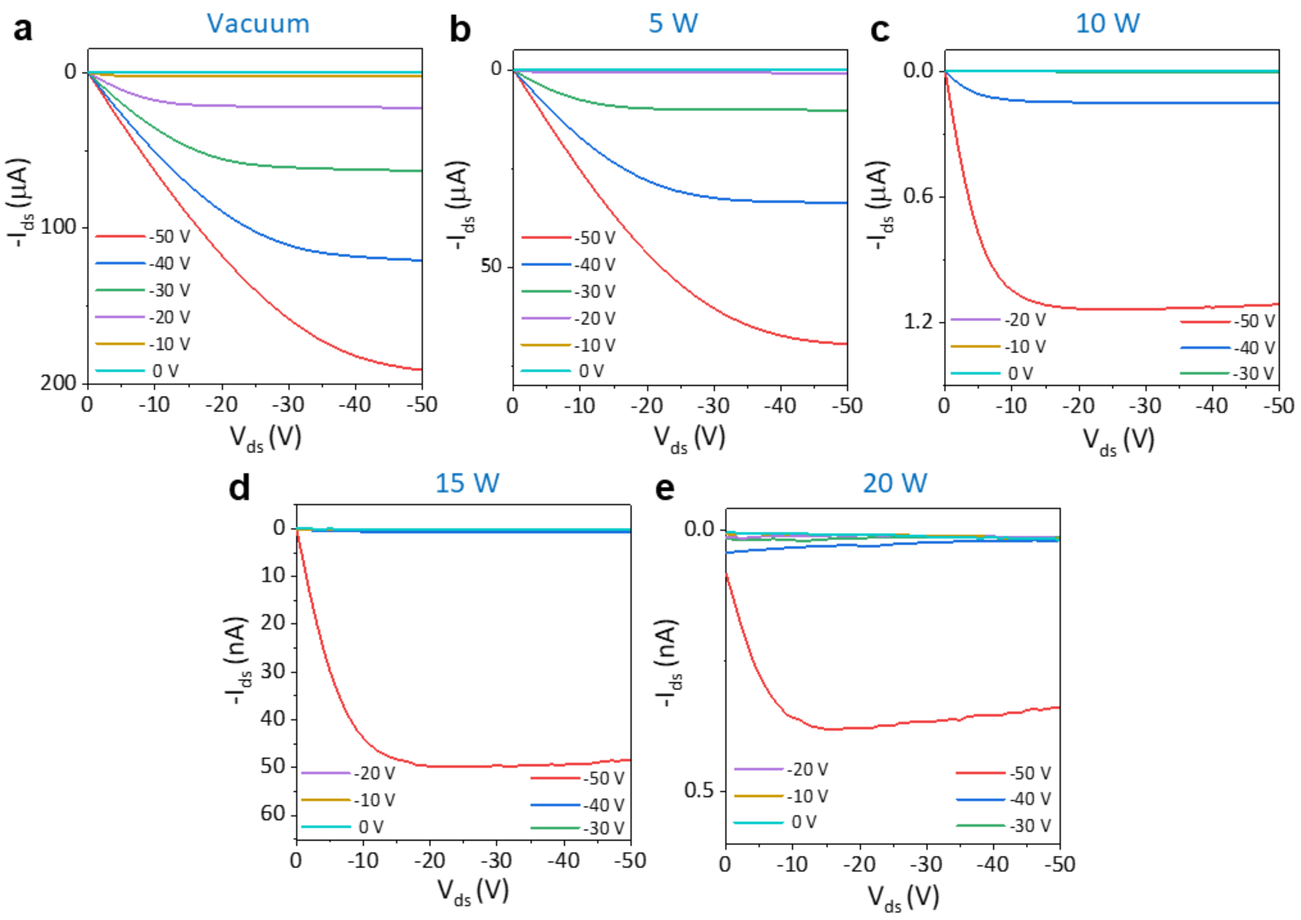

Fig. S10. Evolution of the output curves of the DNTT OFET in the processes of de-doping (a-e). The DNTT OFET shows good output characteristic curves for 6 h in a high vacuum of 10^{-4} Pa (a). After the soft plasma treatment for a short period, the output current decreases (b). With the increase of the de-doping plasma power, the output current continuously decreases, and the pinch-off point becomes noticeably smaller (c-e), suggesting conductivity and threshold voltage deterioration. The bias stress is observed (d and e), which indicates the increase of the density of trap states.

Section 9. General applicability of the de-doping and re-doping methods

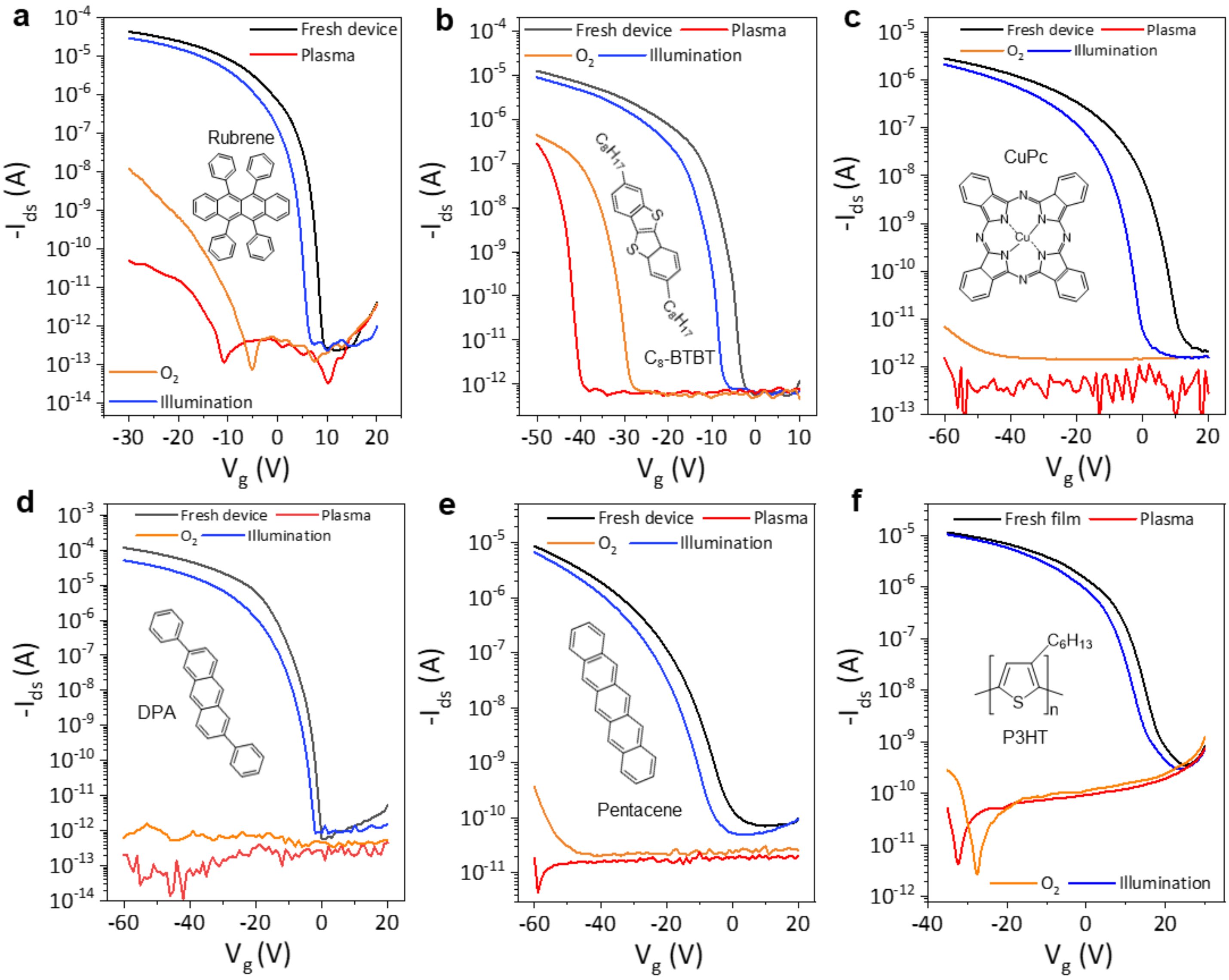

Fig. S11. Molecular universality of the de-doping and re-doping phenomenon. a-f, Transfer curves in the saturation region of the OFET of rubrene (single crystal), C_8-BTBT (crystalline film), CuPc, DPA, pentacene and P3HT in the de-doping and re-doping process. The insets are the corresponding molecular structures. All the OFETs of these semiconductors, with different device structures (top and bottom contact) and aggregate states (single crystal and film), show the same phenomena as the DNTT OFET (**Fig. 2f** and **3a**) in the processes of plasma treatment and illumination in oxygen. These results demonstrate the university of the de-doping and re-doping methods and reveal that OSCs are prevalently doped by oxygen. The incomplete elimination of *p*-type characteristics for single crystal rubrene and the C_8-BTBT crystalline film after plasma treatment may be due to the suppression of the short-circuit diffusion for the plasma and oxygen in thick crystals. The re-doping process of P3HT (<30 min) is much faster than that of small molecules. It may be

attributed to the microstructure difference between polymers and small molecules; oxygen diffusion in highly crystalline small molecules could be slower than disordered polymer chains.

Section 10. Output characteristics in the processes of re-doping

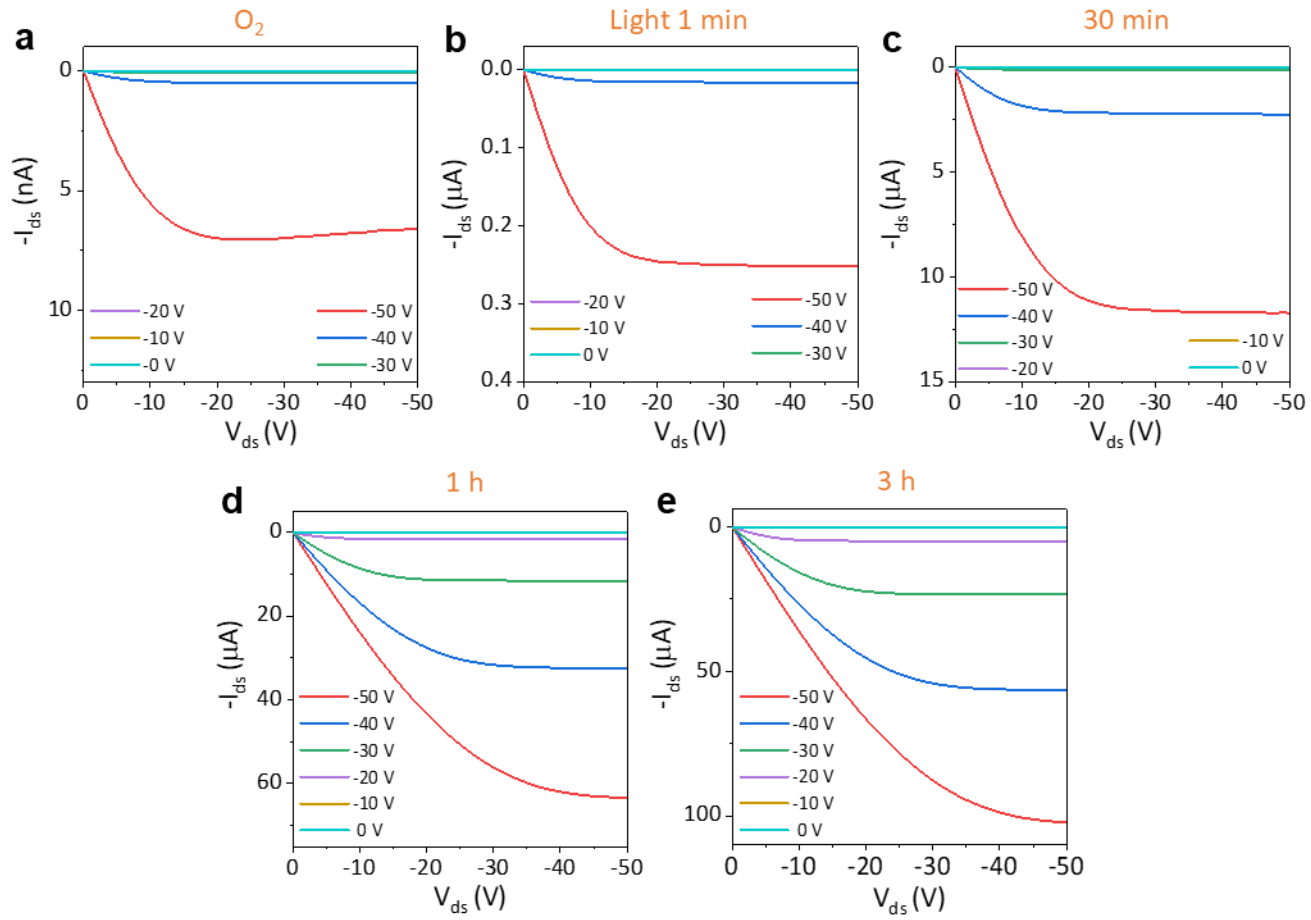

Fig. S12. Evolution of the output curves of the DNTT OFET in the processes of illumination in O_2 (a-e). When 1 atm oxygen is filled in the system for 5 min, the output characteristics of the OFET recover slightly (a). By further extending the time of exposure to oxygen, the rate of recovery is very slow. Interestingly, when the OFET is illuminated by a full-spectrum Xe lamp in an oxygen atmosphere, its output characteristics could recover rapidly, almost to the original level after 3 hours (b-e).

Section 11. Re-doping process under different conditions

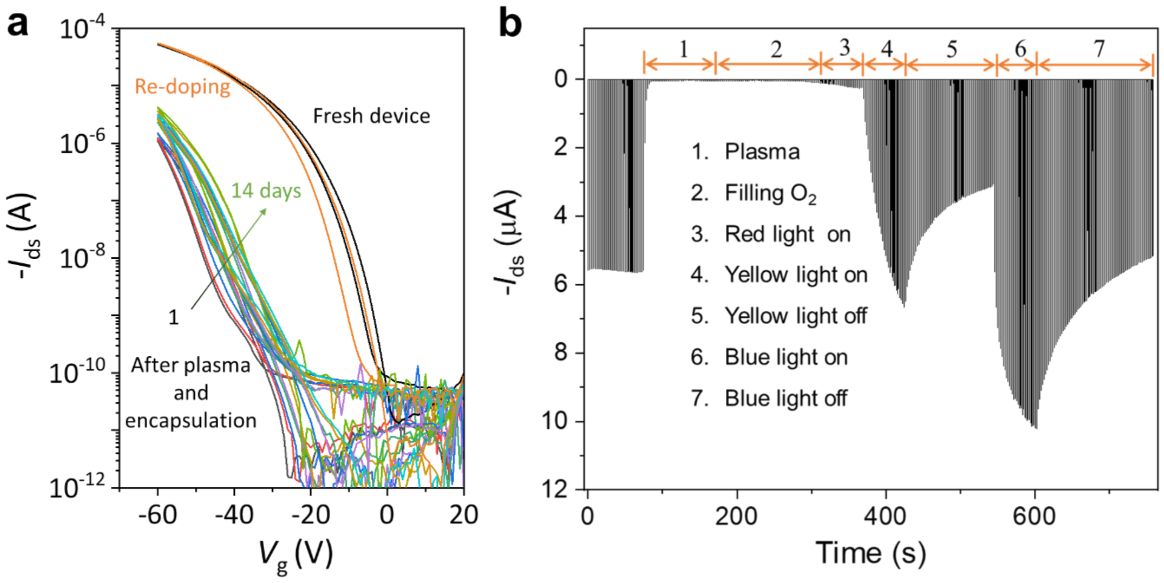

Fig. S13. a, Performance recovery of the DNTT OFET from de-doping state under encapsulation condition. Tthe performance recovery is very slow and limited for 14 days, but it is almost completely recovered when the encapsulation is broken and exposed to light. It indicates that de-doping devices are stable under encapsulation conditions. This result is also consistent with our measurements in inert gases conditions (**Fig. 3c**). **b**, Re-doping DNTT OFETs with different wavelength lights**.** Switching cycle of the ultrathin (about 5 nm) DNTT OFET in above process is measured by a PDA FS380 (semiconductor device analyzers). V_{ds} is set as -30 V and V_{gs} is switched from 0 to -30 V at 1 s intervals. The power of the LED light with different wavelengths is set to 15 W, and exposure time is controlled to be about 1 min. After plasma treatment, the on-state current rapidly decreases. It shows a little recovery under illumination in O_2 with the red light (760~622 nm). After illumination by the yellow light (597~577 nm), the on-state current significantly recovers to half of the original level. Following further illumination with the blue light (450~435 nm), the current almost completely returns to its initial value. The degree of recovery of the DNTT (bandgap absorption of about 450 nm) OFET are blue (450~435 nm) > yellow (597~577 nm) > red (760~622 nm). This implies that bandgap absorption efficiently produces excitons and thus is more beneficial to the re-doping process.

Section 12. Proposed de-doping and re-doping routes

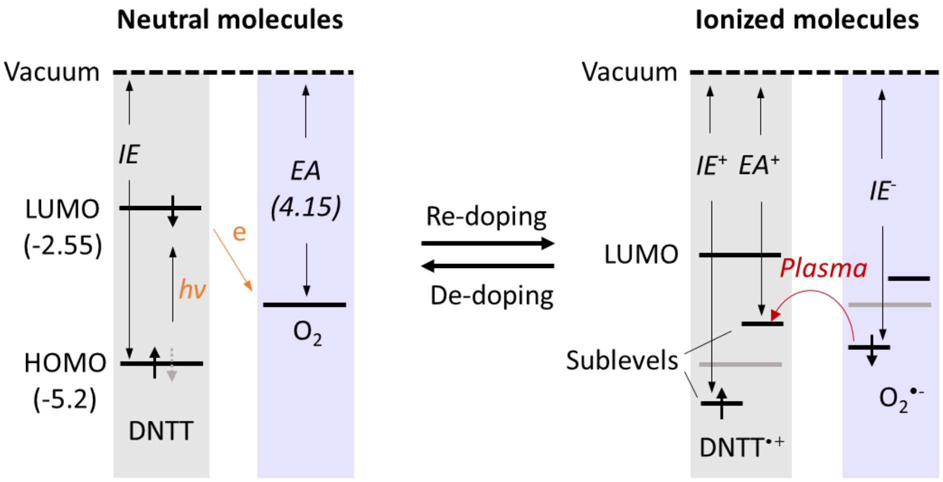

Fig. S14. Proposed de-doping and re-doping routes. DNTT as a representative OSC to describe these processes. The HOMO level (about −5.2 eV, numerically equal to ionization energy, *IE*) of DNTT is estimated by the equation $E_{HOMO} = 21.22 - (E_{cut\text{-}off} - E_F)$ from Ultraviolet Photoelectron Spectroscopy (UPS). The LUMO level (about −2.55 eV, numerically equal to electron affinity, *EA*) is estimated by subtracting the optical band gap (E_g is obtained from the UV-Vis absorption spectrum) from the HOMO level. *EA* of O_2 (*i.e.*, the reduction potential of $O_2/O_2^{\bullet-}$) is about -4.15 eV[19,20].

The deductive re-doping route is that an OSC molecule is photoexcited, *i.e.*, an electron is excited from the HOMO to the LUMO, and then the electron in the excited state is energetic enough to be transferred to a nearby interstitial oxygen molecule. As a result, the neutral organic molecule is oxidized to an organic radical cation (ORC), and O_2 is reduced to a superoxide anion (O_2^-). Given that an organic molecule responds to charge transfer with bond-length changes and a concomitant stabilization of the frontier molecular orbital levels by the relaxation energy, the *IE* and *EA* of an ORC (*i.e.*, IE^+ and EA^+) are larger than that of a neutral molecule[1]. This splits the occupied and unoccupied HOMO-derived sublevels of an ORC. In analogy, the *IE* of O_2^- (IE^-) is larger than that of a neutral molecule, while the *EA* of O_2^- (EA^-) is reverse. For concision, the *IE* of O_2 is not showed. The route of the de-doping is that the unpaired electron of O_2^- returns to ORC, with the energy barrier to charge transfer overcome by the plasma. The bombardment of ions with electric charges is more likely to disrupt the Coulomb interaction of organic semiconductors with oxygen (*i.e.*, organic radical cation and $O_2^{\bullet-}$) than heating. Plasma not only collides with organic molecules to transfer energy efficiently but also transfers charges with organic molecules, while heating transfers energy by phonon vibration. This process generates the neutral semiconductor and an oxygen molecule or other oxygen-related species that escapes from the lattice through diffusion, which is analogous to the removal of H complexed with Mg in GaN by a low-energy electron beam[21].

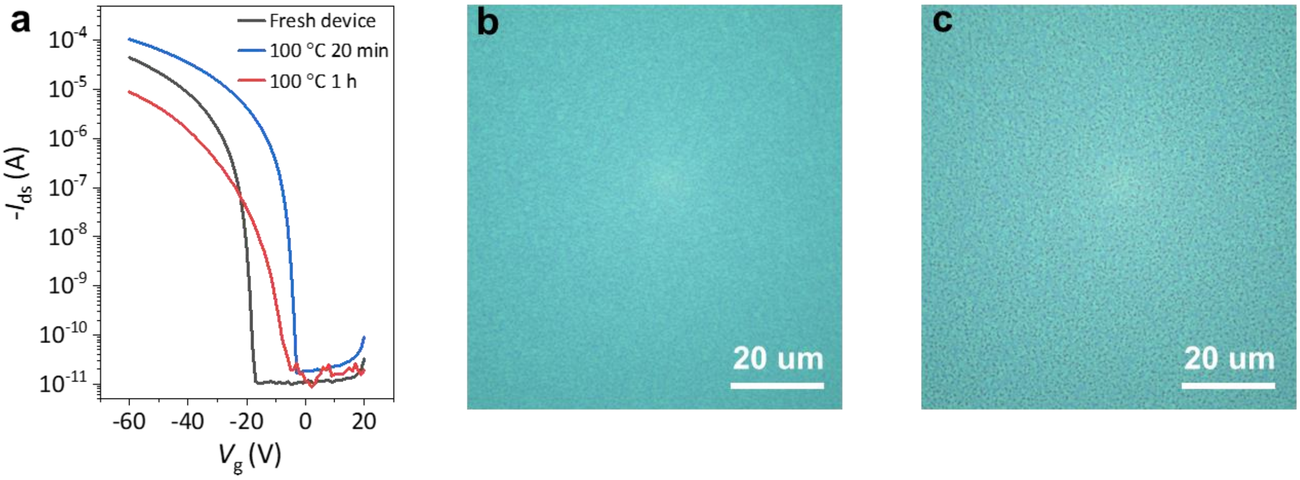

Fig. S15. **a**, Transfer curves of DNTT OFET with different annealing conditions. Images of the DNTT films after annealing 20 min (**b**) and 1 h (**c**) by laser scanning confocal microscope.

The de-doping process can not be accomplished by thermal annealing. As shown in Fig. S15, the DNTT OFETs are thermal annealed of under N_2. The temperature is set as 100 °C because high temperature could lead to rapid film wetting. The performance improvement of DNTT OFET can be observed after annealing 20 min, which is attributed to crystallinity enhancement. However, long annealing time significantly decreases the performance. This decrease in performance is attributed to morphology change caused by the migration of organic molecules[22] and thus is irreversible even under illumination in O_2.

Section 13. Significant improvement of electron transport in *n*-type organic semiconductors.

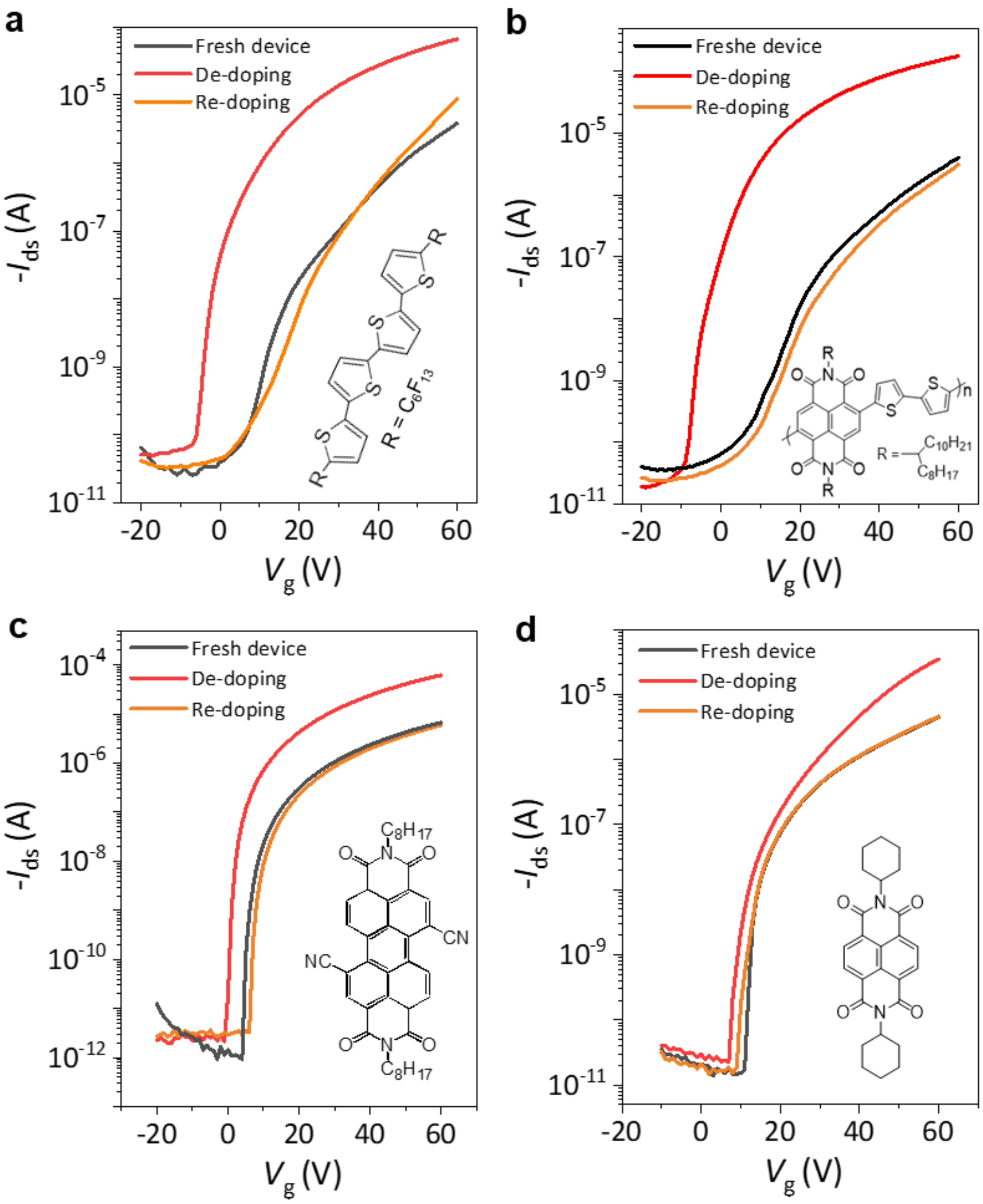

Fig. S16. Transfer curves of DHF-4T (a), N2200 (b), PDI-CN2 (c) and NDI-cy6 (d) OFETs in the processes of de-doping and re-doping. The electron transport of the OFETs is significantly enhanced *via* de-doping. The electron mobilities, threshold voltages and I_{on}/I_{off} rates of these OFETs improve significantly after plasma treatment. When illuminated by a Xe lamp in oxygen, these key performance parameters obviously deteriorate. These results provide a clear explanation for the poor performances of many *n*-type materials and a blueprint for the advance of the performance, stability, and technologies of *n*-type materials and devices.

Section 14. Mobility and threshold voltage statistic for several organic semiconductors.

Table S2. Mobility and threshold voltage statistic for several organic semiconductors in the doped and de-doped states.

	P-type							N-type				
Material	DNTT	Rubrene	C_8-BTBT	CuPc	DPA	Pentacene	P3HT	PDI-C_8	DHF-4T	N2200	PDI-CN	NDI-Cy6
μ_{doped} ($cm^2V^{-1}s^{-1}$)	1.1	3.9	2.9	0.03	1.9	0.28	0.09	0.05	0.04	0.07	0.1	0.07
$\mu_{de\text{-}doped}$	0.6	0.03	1.2	0.02	1.1	0.1	0.04	2.2	0.65	0.78	0.9	0.8
$V_{th\ doped}$ (V)	-3	4	-9	2	-7	-20	17	16	28	28	9	20
$V_{th\ de\text{-}doped}$	-90	-10	-39	-70	-95	-100	-42	9	5	1	4.5	28*

*The transfer curve ofNDI-Cy6 exhibit a nonlinear behavior after de-doping.

The mobility and threshold voltage of the organic semiconductors in the doped and de-doped states are extracted with the large overdrive voltages. The samples are treated by 50 W H_2 plasma for 1 min. As expected, the decrease in mobility of the most p-type OSCs is not very significant, which indicates that mobility is a nature of materials and not significantly influenced by doping even considerable trap states inherent in OSCs. The mobility of rubrene drastically decreased, which may be due to the air gate structure of the rubrene transistor. The n-type materials generally exhibit remarkable increase in mobility with the improved threshold voltages, suggesting that oxygen has a very severe suppress of electron transport.

Section 15. Modulation of conductivity of organic semiconductor.

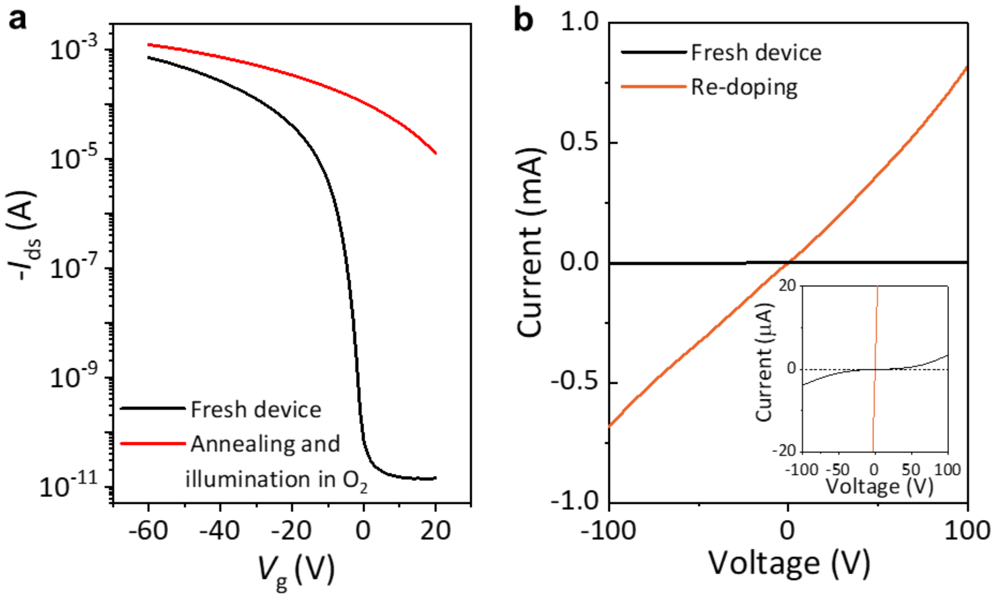

Fig. S17. Transfer curves of the C_{10}-DNTT OFET before and after annealing and illuminating in O_2. (**a**) Transfer curves. (**b**) I-V curves of the C_{10}-DNTT film showing increased conductivity by doping. The inset is the enlarged view of (b). After simultaneous annealing (80 °C) and illumination in 3 atm of oxygen for 3 h, the C_{10}-DNTT OFET changes from accumulation mode to depletion mode and shows weak dependence on gate voltage. The field-effect mobility (μ) shows only a slight decrease (5.2 to 3.6 $cm^2 \cdot V^{-1} \cdot s^{-1}$).

Section 16. Interpretation of the elusive observation of subthreshold swing

The ionization gauge is very commonly used in vacuum system and usually works at the pressure of < 1 Pa. However, everyone has overlooked that the effect of the ionization gauge is as the same as a thermal plasma source. The decrease in conductivity and the shift of threshold voltage of OFETs from atmosphere to vacuum are always attributed to the oxygen desorption by high vacuum. In fact, if the ionization gauge is shut down, the conductivity or threshold voltage of OFETs show no change even under UHV conditions. Accordingly, the plasma caused by ionization gauge is the reason for de-doping of the chemical adsorbed oxygen, *i.e.*, $O_2^{\bullet -}$, rather than vacuum.

The deterioration of subthreshold swing (S) at low temperature[23,24] is previously unclear. Ideally, S should improve at low temperature due to the relationship: $S \equiv (ln10)\frac{dV_{\mathrm{g}}}{d(lnI_{\mathrm{ds}})} = (ln10)\left(\frac{C_{\mathrm{i}}+C_{\mathrm{t}}}{C_{\mathrm{i}}}\right)\frac{kT}{q}$, where C_i and C_t are capacitances of dielectric layer and traps, respectively[10]. However, experimentally, the S value always increases at low temperature in OFETs. This can be explained by the freeze-out effect. At low temperature, the carriers are frozen around the impurities (*i.e.*, impurities are not ionized), and thus the trap states passivated by oxygen doping recover the ability of trapping field-induced carriers, which increases the trap capacitance and thus deteriorates S.

References and Notes

1 Salzmann, I. et al. Molecular electrical doping of organic semiconductors: Fundamental mechanisms and emerging dopant design rules. *Acc. Chem. Res.* **49**, 370-378 (2016).

2 Kang, K. et al. 2d coherent charge transport in highly ordered conducting polymers doped by solid state diffusion. *Nat. Mater.* **15**, 896-902 (2016).

3 Boguslavskii, E. G., Prokhorova, S. A. & Nadolinnyi, V. A. Evolution of ordered films of copper phthalocyanine according to epr data. *J. Struct. Chem.* **46**, 1014 (2005).

4 Podzorov, V. et al. Intrinsic charge transport on the surface of organic semiconductors. *Phys. Rev. Lett.* **93**, 086602 (2004).

5 Kalb, W. L., Meier, F., Mattenberger, K. & Batlogg, B. Defect healing at room temperature in pentacene thin films and improved transistor performance. *Phys. Rev. B* **76**, 184112 (2007).

6 Diemer, P. J. et al. Quantitative analysis of the density of trap states at the semiconductor-dielectric interface in organic field-effect transistors. *Appl. Phys. Lett.* **107** (2015).

7 Burlingame, Q. et al. Centimetre-scale electron diffusion in photoactive organic heterostructures. *Nature* **554**, 77-80 (2018).

8 Fratini, S. et al. A map of high-mobility molecular semiconductors. *Nat. Mater.* **16**, 998-1002 (2017).

9 Lee, B. et al. Trap healing and ultralow-noise hall effect at the surface of organic semiconductors. *Nat. Mater.* **12**, 1125-1129 (2013).

10 Sze, S. M. & Ng, K. K. *Physics of semiconductor devices*. John wiley & sons, 2006.

11 Tietze, M. L. et al. Doped organic semiconductors: Trap-filling, impurity saturation, and reserve regimes. *Adv. Funct. Mater.* **25**, 2701-2707 (2015).

12 Borchert, J. W. et al. Small contact resistance and high-frequency operation of flexible low-voltage inverted coplanar organic transistors. *Nat. Commun.* **10**, 1119 (2019).

13 Kanagasekaran, T. et al. A new electrode design for ambipolar injection in organic semiconductors. *Nat. Commun.* **8**, 999 (2017).

14 Betti, M. G. et al. Barrier formation at organic interfaces in a cu(100)-benzenethiolate-pentacene heterostructure. *Phys. Rev. Lett.* **100**, 027601 (2008).

15 Liu, Z. et al. Contact engineering for organic semiconductor devices via fermi level depinning at the metal-organic interface. *Phys. Rev. B* **82**, 035311 (2010).

16 Oehzelt, M., Koch, N. & Heimel, G. Organic semiconductor density of states controls the energy level alignment at electrode interfaces. *Nat. Commun.* **5**, 4174 (2014).

17 Lee, S. & Nathan, A. Subthreshold schottky-barrier thin-film transistors with ultralow power and high intrinsic gain. *Science* **354**, 302-304 (2016).

18 Jiang, C. et al. Printed subthreshold organic transistors operating at high gain and ultralow power. *Science* **363**, 719-723 (2019).

19 Tang, C. G. et al. Doped polymer semiconductors with ultrahigh and ultralow work functions for ohmic contacts. *Nature* **539**, 536-540 (2016).

20 Kong, J. et al. Co_2 doping of organic interlayers for perovskite solar cells. *Nature* **594**, 51-56 (2021).

21 Nakamura, S. The roles of structural imperfections in ingan-based blue light-emitting diodes and laser diodes. *Science* **281**, 956-961 (1998).

22 Chen, X. et al. Balancing the film strain of organic semiconductors for ultrastable organic transistors with a five-year lifetime. *Nat. Commun.* **13**, 1480 (2022).

23 Mei, Y. et al. Crossover from band-like to thermally activated charge transport in organic transistors due to strain-induced traps. *Proc. Natl. Acad. Sci. U.S.A.* **114**, E6739-E6748 (2017).

24 Minari, T., Nemoto, T. & Isoda, S. Temperature and electric-field dependence of the mobility of a single-grain pentacene field-effect transistor. *J. Appl. Phys.* **99**, 034506 (2006).
